# DNA methylation enzymes in the kidneys of male and female BTBR ob/ob mice

**DOI:** 10.3389/fendo.2023.1167546

**Published:** 2023-04-05

**Authors:** Beatriz Maria Veloso Pereira, Mariana Charleaux de Ponte, Ana Paula Malavolta Luz, Karina Thieme

**Affiliations:** Laboratório de Bases Celulares e Moleculares da Fisiologia Renal, Departamento de Fisiologia e Biofísica, Instituto de Ciências Biomédicas, Universidade de Sao Paulo, Sao Paulo, SP, Brazil

**Keywords:** epigenetics, DNMT, TET, chronic kidney disease, obesity and diabetes

## Abstract

Diabetic kidney disease (DKD) is the leading cause of the end-stage renal disease. Recent studies have shown that epigenetic modifications contribute to alterations in gene expression and the development of DKD. This study aimed to show an expression profile of key DNA (de)methylation enzymes (DNMT, TET proteins) and their differences between sexes under obesity and diabetic condition. Male and female black and tan brachyury (BTBR) ob/ob mice and their corresponding wild-type littermates (BTBR WT) were studied until 16 weeks of age. Metabolic parameters, kidney morphophysiology and the expression of fibrotic markers and epigenetic enzymes were studied in whole kidney tissue or specifically in the glomerulus. The results showed sexual dimorphism in the development of metabolic disease and in kidney morphophysiology. Female mice have a different profile of DNMTs expression in both WT and obese/diabetic condition. Furthermore, metabolic condition negatively modulated the glomerular expression of TET1 and TET3 only in females. To our knowledge, this is the first study that shows a kidney profile of the expression of key (de)methylation enzymes, DNMTs and TETs, in the BTBR ob/ob experimental model of DKD and its association with sex. The knowledge of this epigenetic profile may help future research to understand the pathophysiology of DKD in males and females.

## 1 Introduction

In recent years, the incidence of chronic kidney disease (CKD) has grown faster and in an astounding way. The number of deaths caused by CKD has nearly doubled since 1990, reaching the 12th leading cause of death in 2017 (1). Moreover, studies suggest that this position will ascend to fifth place by 2040 (2, 3). The last Global Burden of Disease study has shown that CKD has a higher prevalence in females and higher mortality in males, which suggests that males progress to end-stage kidney disease more rapidly (4). However, this data also highlights that a better characterization of sex-specific disease markers and the underlying pathophysiological mechanisms is still needed.

Diabetic kidney disease (DKD) is a multifactorial chronic disorder that arises as a complication in patients with type 1 and 2 diabetes. In fact, DKD is the most common cause of renal failure worldwide and although not all diabetic patients develop nephropathy, type 2 diabetes remains the primary cause of DKD. Its prevalence is estimated to affect more than 783 million people by 2045, which represents 12% of the world’s population (5).

Chronic hyperglycemia induces dysfunction in various cell types of the kidney leading to albuminuria and progressive decline in organ function, which are associated with glomerular damage and tubule-interstitial fibrosis (6–8).

Although recent advances have been made in the understanding of the pathophysiology and treatment of DKD, the adaptation of experimental innovations to clinical reality is one of the greatest challenges. Furthermore, the understanding of the molecular mechanisms that induce the set of pathologic and clinical alterations that determine DKD in humans is still deficient (9).

The term epigenetics refers to chromatin modifications that are heritable and induce changes in gene expression without alterations in the classical DNA base code. Epigenetic modifications can profoundly affect the expression of nearby genes by altering chromatin structure, affecting transcription factor binding, or influencing DNA methylation (7).

DNA methylation in mammals involves the addition of a methyl group at position 5 of cytosines located mostly on CpG islands in the promoter regions of the genes, by enzymes called DNA methyltransferases (DNMT), forming 5-methylcytosine (5-mC). DNMT1 is considered a maintenance methyltransferase, that copies the methylation pattern of the parental DNA strand conserving it during replication. On the other hand, DNMT3A and DNMT3B are *de novo* methyltransferases, whose function establish new DNA methylation patterns. Hypermethylation of gene promoter is associated with gene expression repression and can be reversed both passively or actively (10). Passive demethylation occurs by progressive “dilution” of 5-mC over mitosis. In this case, DNMT1 is excluded from the replication fork and the newly synthesized DNA strand is not methylated. On the other hand, active demethylation occurs by enzymatic removal of methyl cytosines (11). Active demethylation depends on enzymes called Ten Eleven Translocation (TET). The TET family consists of three members (TET1, TET2 and TET3), which catalyze the formation of a hydroxy-methylated cytosine (5-hmC). The addition of this hydroxyl group to methylated DNA is associated with gene expression activation (12, 13).

It is known that a wide range of epigenetic modifications including DNA methylation influence gene expression in DKD, causing an impact on the progression of kidney injury and loss of function (14, 15). However, the expression of DNA methylation enzymes in renal tissue in DKD and the influence of sex on this profile are still not fully understood. Thus, this study aimed to investigate the expression of (de)methylation enzymes in the kidneys of both male and female BTBR ob/ob mice, which are obese, hyperglycemic, and hypertriglyceridemic, representing a gold standard model of DKD. The results presented here intend to contribute to the knowledge about the association of epigenetic modification players and the progression of DKD in both sexes.

## 2 Materials and methods

### 2.1 Animals

All procedures regarding animal experimentation were approved by the Animal Use Ethics Committee of the Institute of Biomedical Sciences, University of São Paulo (numbers 5348280918 and 7358100320). Male and female black and tan brachyury (BTBR) ob/ob mice and their corresponding wild-type littermates (BTBR WT) were studied until 16 weeks of age (total of 28 mice). Breeding pairs BTBR/ob heterozygotes (BTBR.Cg-Lepob/WiscJ; stock no. 004824) were purchased from Jackson Laboratories (Bar Harbor, ME USA) and maintained at the experimental facility of the Department of Pharmacology of the Institute of Biomedical Sciences of the University of São Paulo, under a light-dark cycle and free access to food and water.

### 2.2 Metabolic and biochemical parameters

Body weight and blood glucose levels were collected every four weeks to follow the progress of obesity and diabetes. Mice were fasted for two hours before the blood glucose measurements (Accu-Chek Performa Kit, Sao Paulo, SP, Brazil) (16). Six hours before euthanasia, the animals were housed in metabolic cages for urine sample collection. Urine creatinine levels were evaluated using a colorimetric test (Labtest, Lagoa Santa, MG Brazil) according to the manufacturer’s instructions (17). Albuminuria was detected using an ELISA kit (Assaypro, Saint Charles, MO USA), conforming to the manufacturer’s recommendations. Additionally, we submitted creatinine-corrected urine samples on a 10% polyacrylamide gel, which was subsequently subjected to silver staining using SilverQuest Silver Staining Kit (Sigma-Aldrich, Saint Louis, MO USA) (18). Albumin-corresponding bands were analyzed using the ImageJ software (National Institutes of Health, Bethesda, MD USA) and the results are presented as arbitrary units.

### 2.3 Kidney tissue histology and immunostaining

4 µm slices of paraformaldehyde-fixed and paraffin-embedded kidney tissue were stained with periodic-acid Schiff (PAS) for evaluation of glomerular morphology. The total area and the PAS-positive stained area of at least 15 glomeruli per animal were analyzed using NIS-Elements software (Nikon, Minato, TO Japan) coupled to a light microscope (Eclipse 80i) and the ImageJ software.

For immunofluorescence studies, kidney slices were deparaffinized and submitted to antigenic retrieval with EDTA buffer (1mM EDTA, 0.05% Tween 20, pH 8) (19, 20). After blocking (Protein Block, DAKO Agilent Technologies Inc., Saint Clair, CA USA) for 1 hour, sections were incubated overnight at 4° C using the following primary antibodies: mouse anti-collagen IV (1:400, Abcam, Cambridge, UK); rabbit anti-TET1 (1:150, Genetex, Radnor, PA USA); rabbit anti-TET3 (1:100, Novus Biotechnology, Centennial, CO USA); and rabbit anti-Wilms’ tumor suppressor 1 (WT1) (1:200, Novus Biotechnology). On the next day, after washing with phosphate-buffered saline (PBS) 3 times of 5 minutes, slices were incubated with Alexa Fluor^®^ 488 goat anti-rabbit or anti-mouse (1:200) or Rhodamine Red™-X goat anti-rabbit (1:200) (Jackson ImmunoResearch Laboratories, Pennsylvania, PA USA). Slides stained for WT1 were also incubated with 4′,6-diamidino-2-phenylindole (DAPI) for nuclear staining (1:20000, Sigma-Aldrich). For glomerular TETs staining quantification, the sum of the gray values of all pixels divided by the number of pixels of the glomerular area was performed. For glomerular collagen IV staining quantification, the following formula was used: total fluorescence = integrated density - (selected glomerular area x mean background fluorescence). For WT1 staining quantification, positive nuclei were counted per glomerulus. At least 15 glomeruli per mouse were analyzed in a blinded manner and quantifications were performed using ImageJ software.

For the immunohistochemistry studies, slices were deparaffinized and antigen retrieval was performed as previously described for immunofluorescence staining. After blocking, the slices were incubated overnight at 4° C with rabbit anti-DNMT3a (1:100, Cell Signaling Technology Inc., Danvers, MA USA) and, on the next day, after washing with PBS 3 times of 5 minutes, sections were incubated with the EnVision System HRP Labeled Polymer anti-rabbit (DAKO Agilent Technologies). 3, 3′ diaminobenzidine tetrahydrochloride (DAB) substrate (DAKO Agilent Technologies) was used to produce a dark brown precipitate over the antigen-antibody site. Counterstaining was performed with Hematoxylin (EasyPath, Indaiatuba, SP Brazil). Glomerular DNMT3a staining was analyzed using NIS-Elements software coupled to a light microscope and the ImageJ software. For DNMT3a quantification, we used optical density (OD), which is the result of the following equation: OD = log(max intensity/mean gray intensity), where max intensity = 255 for 8-bit images. At least 40 glomeruli per mouse were analyzed.

### 2.4 Quantitative real-time PCR

Snap-frozen kidney tissue was pulverized in liquid nitrogen and RNA isolation was performed with TRIzol^®^ reagent (Invitrogen, Waltham, MA USA). Reverse transcription was conducted using High-Capacity RNA-to-cDNA kit (Applied Biosystems, Foster City, CA USA), according to the manufacturer’s protocol. Real-time PCR (RT-PCR) was performed using *TaqMan* probes on the StepOne system (Applied Biosystems). The data were analyzed using the comparative cycle threshold (2^ΔΔCt^) method. The results were normalized by *Ppia* expression and are shown as *fold change* relative to the male BTBR WT group. The following *TaqMan* probes were used: *Ppia*, Mm02342430_g1; *Col4a1*, Mm01210125_m1; *Tgfb1*, Mm01178820_m1; *Tgfbr1*, Mm00436964_m1; *Tgfbr2*, Mm03024091_m1; *Tet1*, Mm01169087_m1; *Tet2*, Mm00524395_m1; *Tet3*, Mm00805756_m1; *Dnmt1*, Mm01151063_m1; *Dnmt3a*, Mm00432881_m1; and *Dnmt3b*, Mm01240113_m1.

### 2.5 Immunoblotting

Kidney tissue samples were homogenized with Cell Lysis Buffer (Cell Signaling Technology) and submitted to sonication. Protein quantification was performed using bicinchoninic acid assay (Sigma-Aldrich). Then, 50 μg of protein were added to a 10% polyacrylamide gel and separated by electrophoresis. Samples were transferred to nitrocellulose membranes (GE Healthcare, Chicago IL USA), blocked in 5% non‐fat milk diluted in TBS‐T buffer (Tris 50 mM, NaCl 150 mM, and Tween-20 0.1%), and subsequently incubated with primary antibodies (TGF-β1 – 1:1000, Novus Biotechnology; and GAPDH – 1:1000, Cell Signaling Technology) overnight at 4°C. On the next day, after washing with TBS-T, membranes were incubated with conjugated goat secondary antibodies against rabbit (Cell Signaling Technology) and were exposed to the ECL™ luminescence reagent (GE Healthcare, Chicago, IL USA) for immunodetection in the Amersham Imager 600 (GE Healthcare). The quantification of the blots was performed using the ImageJ software. Results were normalized by GAPDH expression and presented as protein expression relative to the male BTBR WT group.

### 2.6 Statistical analysis

Data analysis was performed using two-way ANOVA for sex and metabolic conditions (obesity and diabetes). When the interaction between these factors was significant, Tukey’s multiple comparisons test was performed. All statistical analysis was performed using GraphPad Prism 8.0 software (GraphPad Software, Inc., San Diego, CA USA) and results are presented as the mean ± SD. *p<0.05* was considered statistically significant.

## 3 Results

### 3.1 The development of obesity and diabetes is different between males and females

**Figure 1:**
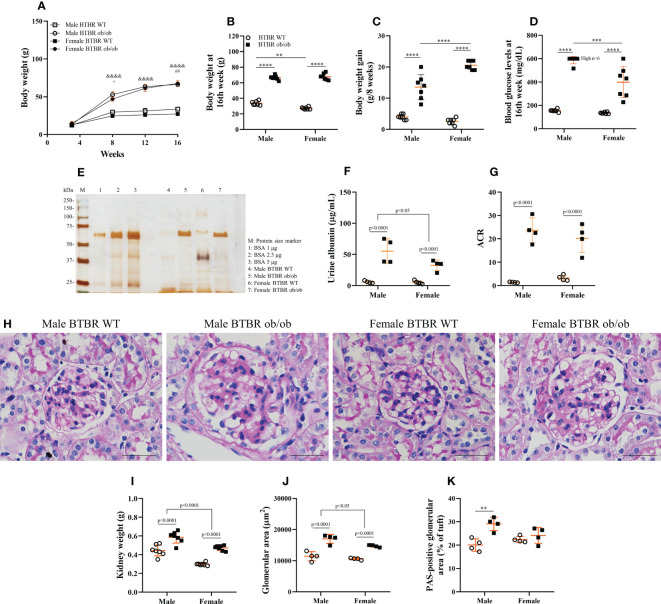
The development of obesity and diabetes and their effect on kidney function and morphology is different between males and females. Effects of sex and metabolic condition in BTBR mice on body weight/week **(A)**, body weight at 16^th^ week **(B)**, body weight gain **(C)**, blood glucose levels at 16^th^ week **(D)**, urine albumin **(E, F)**, albumin to creatinine ratio **(G)**, kidney weight **(H)**, glomerular area **(I)**, PAS-positive glomerular area **(J)**, and glomerular morphology **(K)**. *p* values corresponding to the independent effect of metabolic condition or sex are specified in the graphs. *p* values of two-way ANOVA followed by Tukey’s multiple comparisons test are as follows: ^&&&&^p<0.0001 *versus* the respective WT controls; ^+^p<0.05 *versus* male BTBR ob/ob; ^##^p<0.01 *versus* male BTBR WT. The values are the mean ± SD (n = 4 - 7). **(E)**: Representative full-length silver-stained gel of urine samples. **(K)**: Kidney slices were stained with Periodic acid–Schiff for morphological analysis and images were captured using NIS-Elements software coupled to a light microscope equipped with a 40x objective. Bars = 50 μm. WT, wild type; kDa, protein size; BSA, bovine serum albumin; ACR, albumin to creatinine ratio. **p< 0.01; ***p< 0.001, ****p<0.0001.

To assess whether the progression of obesity and diabetes differs between sexes, metabolic parameters were analyzed every four weeks. As soon as 8 weeks old, BTBR ob/ob mice showed increased body weight in comparison to the respective WT, although obese female mice showed decreased body weight in comparison to obese males (**Figure 1A**). However, this sex difference was no longer seen at 16 weeks, with female and male obese mice showing similar body weights (**Figure 1B**). Still, the overall gain weight of female obese mice was higher than male obese mice (**Figure 1C**). Despite the final obesity level, female BTBR ob/ob mice were less hyperglycemic than males at the end of the protocol (**Figure 1D**).

### 3.2 Sex and metabolic condition affect differently kidney function and morphology

**Figure 2:**
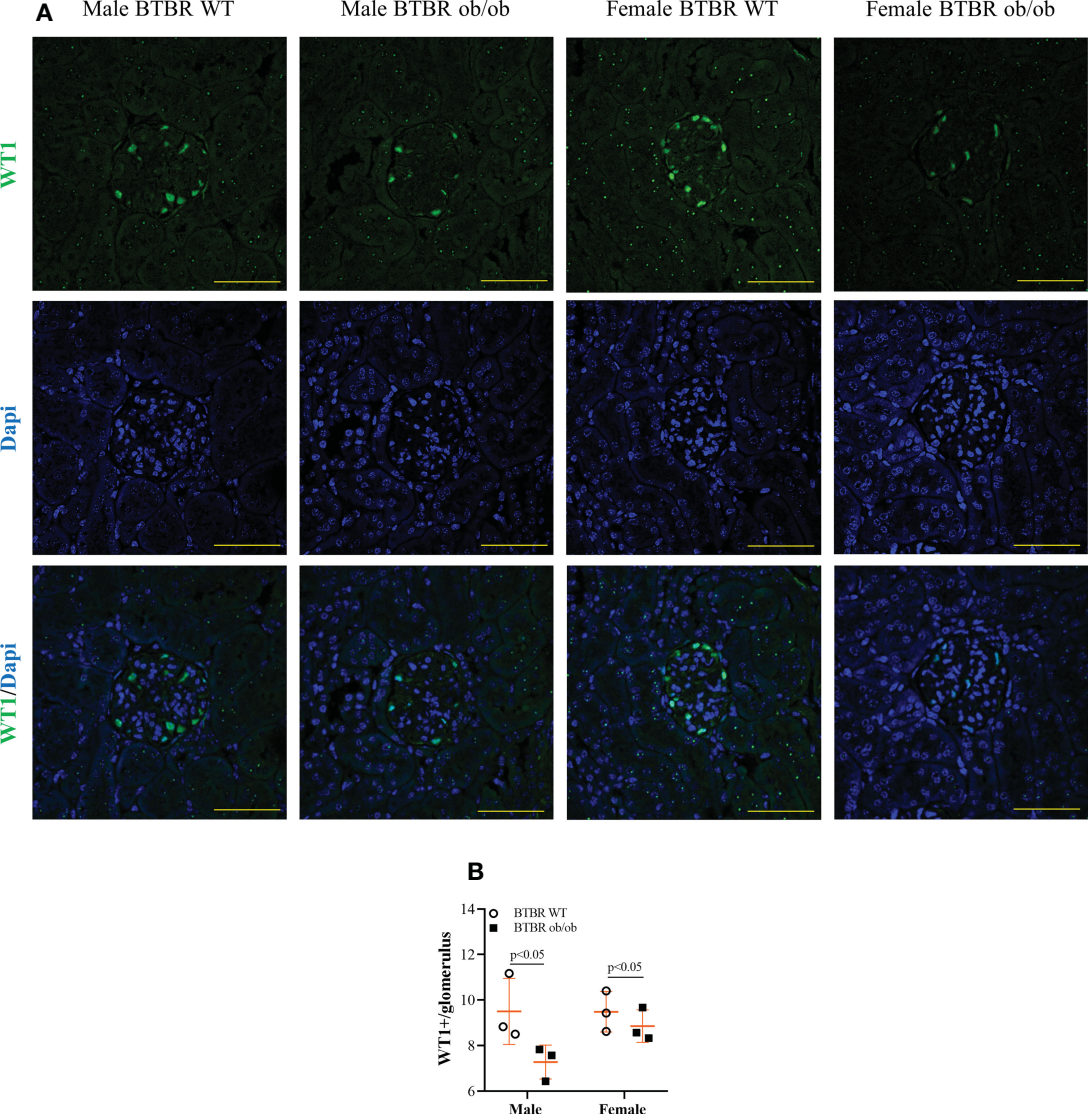
Obesity and diabetes reduce WT1 positive cells in both males and females. Effect of sex and metabolic condition on WT1 positive cells per glomerulus. Representative images of WT1 and DAPI staining **(A)** and glomerular WT1 positive cells quantitation **(B)**. The values are the mean ± SD (n = 3). Immunofluorescence images were captured using NIS-Elements software coupled to a light microscope equipped with a 40x objective at laser excitation of 488 nm (Eclipse 80i). Bars = 50 μm. WT, wild type. *p* values corresponding to the independent effect of metabolic condition or sex are specified in the graphs.

Diabetic milieu contributes to glomerular and tubular cells injury. Indeed, the renal function results showed that obesity and diabetes led to albuminuria and increased albumin-to-creatinine ratio (ACR) in both male and female mice (**Figures 1E–G**). The observed loss of albumin in the urine was accompanied by increased kidney weight (**Figure 1H**), larger glomerular tuft area (**Figure 1I**), and decline in WT1-positive cells (**Figure 2**) which indicates damage in the glomerular epithelial cells named podocytes, with consequent disruption of the glomerular filtration barrier. From these data, it is important to note that an independent effect of sex was also observed on urine albumin, kidney weight, and glomerular area parameters. Furthermore, only male BTBR ob/ob mice displayed a significant increase in the PAS-positive area (**Figures 1J, K**).

### 3.3 Obesity and diabetes increase whole kidney TGF-β1 content and glomerular expression of collagen IV in both males and females

**Figure 3:**
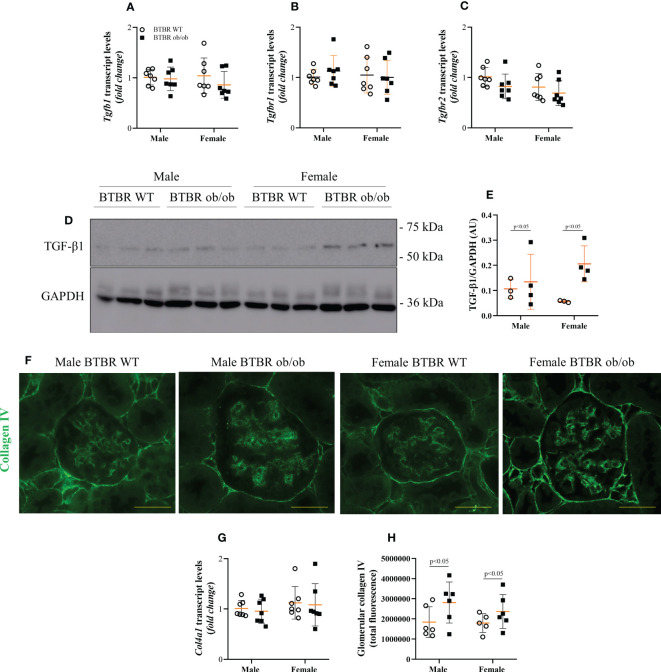
Obesity and diabetes increase whole kidney TGF-β1 content and glomerular expression of collagen IV in both males and females. Effects of sex and metabolic condition in BTBR mice on *Tgfb1* **(A)**, *Tgfbr1* **(B)**, and *Tgfbr2* **(C)** transcript levels, TGF-β1 protein expression **(D, E)**, collagen IV transcript levels **(G)**, and glomerular collagen IV content **(F, H)**. The values are the mean ± SD (n = 3 - 7). Immunofluorescence images were captured using NIS-Elements software coupled to a light microscope equipped with a 40x objective at laser excitation of 488 nm (Eclipse 80i). Bars = 50 μm. WT, wild type; AU, arbitrary units; *Tgfb1*, TGF-β1; *Tgfbr1*, TGF-β receptor 1; *Tgfbr2*, TGF-β receptor 2; *Col4a1*, collagen IV. *p* values corresponding to the independent effect of metabolic condition or sex are specified in the graphs.

Enlargement of glomerular tuft area and increase in PAS-positive area indicate increased matrix deposition. Thus, the expression of fibrotic markers was further investigated. Transcript levels of *Tgfb1, Tgfbr1*, and *Tgfbr2* were not changed between groups (**Figures 3A–C**). However, at the protein level, male and female BTBR ob/ob mice showed higher TGF-β1 protein expression in total kidney tissue (**Figures 3D, E**). As the increase in collagen IV production is one of the effects of TGF-β1 activation, the glomerular expression of collagen IV was also explored. Despite no difference in *Col41a* transcript levels (**Figure 3G**), glomerular protein expression of collagen IV was increased by the metabolic condition in male and female BTBR ob/ob mice (**Figures 3F, H**).

### 3.4 Female BTBR ob/ob mice exhibit decreased glomerular expression of the epigenetic enzyme DNMT3A

**Figure 4:**
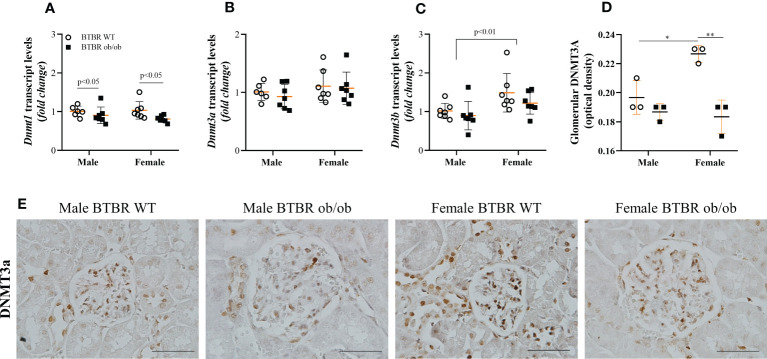
Female BTBR ob/ob mice exhibit decreased glomerular expression of epigenetic enzyme DNMT3A. Effects of sex and metabolic condition in BTBR mice on *Dnmt1* **(A)**, *Dnmt3a* **(B)**, and *Dnmt3b* **(C)** transcripts levels and on total glomerular expression of DNMT3a **(D, E)**. The values are the mean ± SD (n = 3 - 7). Immunohistochemistry images were captured using NIS-Elements software coupled to a light microscope equipped with a 40x objective (Eclipse 80i). Bars = 50 μm. WT, wild type; *Dnmt1*, DNA methyltransferase 1; *Dnmt3a*, DNA methyltransferase 3 alpha; *Dnmt3b*, DNA methyltransferase 3 beta. *p* values corresponding to the independent effect of metabolic condition or sex are specified in the graphs. ^*^p<0.05 *versus* male BTBR WT; ^**^p<0.05 versus female BTBR WT.

Subsequently, the kidney profile of enzymes associated with the state of DNA methylation was investigated. First, the expression of DNMT enzymes, responsible for the formation of 5-mC was analyzed and different patterns for each member of the family were observed. Regarding transcript levels of the genes, BTBR ob/ob mice exhibited decreased *Dnmt1* compared to the respective WT group (**Figure 4A**). Meanwhile, no difference was observed in *Dnmt3a* among groups (**Figure 4B**), but an independent effect of sex was noticed on *Dnmt3b* (**Figure 4C**). The sex effect was also observed when the glomerular protein expression of DNMT3A was explored by immunohistochemistry. Female BTBR WT exhibited increased glomerular expression of DNMT3A in comparison to male BTBR WT (**Figures 4D, E**). Moreover, female BTBR ob/ob mice showed decreased glomerular expression of this enzyme when compared to female BTBR WT, which designates also an effect of the metabolic condition on this parameter (**Figures 3D, E**). Together, these results suggest that female mice have a different profile of DNMTs expression in both WT and obese/diabetic condition.

### 3.5 Obesity and diabetes negatively modulate glomerular TET proteins expression in females

**Figure 5:**
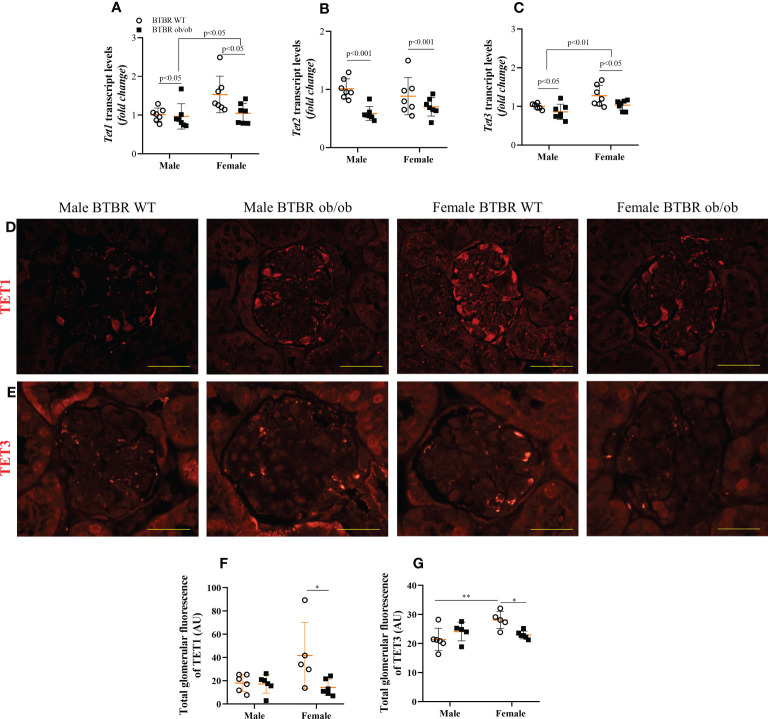
Obesity and diabetes negatively modulate TET protein expression in females. Effects of sex and metabolic condition in BTBR mice on *Tet1* **(A)**, *Tet2* **(B)**, and *Tet3* **(C)** transcript levels and on total glomerular fluorescence of TET1 **(D, F)** and TET3 **(E, G)**. The values are the mean ± SD (n = 5 - 7). Immunofluorescence images were captured using NIS-Elements software coupled to a light microscope equipped with a 40x objective at laser excitation of 543 nm (Eclipse 80i). Bars = 50 μm. WT, wild type; AU, arbitrary units; *Tet1*, Tet Methylcytosine Dioxygenase 1; *Tet2*, Tet Methylcytosine Dioxygenase 2; *Tet3*, Tet Methylcytosine Dioxygenase 3. *p* values corresponding to the independent effect of metabolic condition or sex are specified in the graphs. ^**^p<0.05 *versus* male BTBR WT; ^*^p<0.05 versus female BTBR WT.

When analyzing the demethylation enzymes, independent effects of sex were observed on *Tet1* and *Tet3* transcripts levels, with females exhibiting increased expression in comparison to male mice. The metabolic condition also induced a decrease in the transcript levels of these genes both in male and female mice (**Figures 5A, C**). On the other hand, *Tet2* transcript levels were only modulated by the metabolic condition, with BTBR ob/ob mice showing decreased gene expression (**Figure 5B**). To assess the glomerular protein expression of TET1 and TET3, immunofluorescence experiments were performed (**Figures 5D, E**). There was an interaction of both factors (metabolic condition and sex) in the glomerular expression of TET1 and TET3. Female BTBR ob/ob mice showed decreased total glomerular fluorescence of TET1 and TET 3 compared to the female BTBR WT mice and no differences were observed in male mice (**Figures 5F, G**). Interestingly, as observed for DNMT3A, female WT mice showed an increased expression of TET3 compared to male WT mice, (**Figure 5G**). Together, these data demonstrate that obesity and diabetes negatively modulate the glomerular expression of TET1 and TET3 only in females.

## 4 Discussion

The BTBR *ob/ob* mouse model of DKD offers several important advantages compared with other existing models of diabetes and obesity, as it recapitulates most of the clinical and morphological renal lesions observed in patients with type 2 diabetes (21). The results of the present study confirmed that both male and female BTBR ob/ob mice develop metabolic perturbations including obesity and persistent hyperglycemia. Interestingly, although the final body weight of male and female BTBR ob/ob mice is similar, the progression of the body weight gain is different between both sexes. Furthermore, hyperglycemia is also not as prominent in females as in males. These differences were also observed by Hudkins et al. (21).

The results also confirmed the expected loss of kidney function, albuminuria, and histopathological alterations of this obesity and diabetic model (22, 23). Interestingly, the lower blood glucose levels of female BTBR ob/ob mice compared to male BTBR ob/ob mice reflected in lower urine albumin and as well as attenuated histopathological alterations. Despite the increased kidney weight and glomerular area, female BTBR ob/ob mice did not show increased PAS-positive glomerular area compared to WT group at 16^th^ weeks.

mRNA levels of *Tgfb1* were not increased, as previously observed in the study by Opazo-Rios et al. (22) with 22-week-old BTBR ob/ob mice. Other studies of DKD showed upregulation of *Tgfb1*, associated with increased DNA demethylation. Oba et al. (24) showed in primary mesangial cells of diabetic db/db mice that demethylation of *Tgfb1* DNA and upregulation of *Tgfb1* mRNA progressed simultaneously. They also demonstrated that reactive oxygen species (ROS) play an important role in the aberrant DNA methylation of this gene. ROS overproduction increased the binding of the upstream transcription factor 1 (USF1) to its recognition site in the *Tgfb1* promoter region and decreased binding of DNMT1/DNMT3B, contributing to the enhanced transcription of the *Tgfb1* gene. Another study by Yang et al. (25) explored the role of TET2 on the pathogenesis of DKD through the activation of TGFβ-1 *via* DNA demethylation. They found increased expression of DNMT3B, TET2, and TGFβ-1 in the renal cortex of db/db mice. Although both enzymes related to DNA methylation status were up-regulated, only TET2 recruitment to the *Tgfb1* promoter was increased, contributing to *Tgfb1* demethylation and increased mRNA levels. The difference between the present study and the ones described above could be explained by the time point studied and the diabetic model selected. They also exemplify the complexity and dynamic of diabetes pathogenesis and how epigenetics is an important player in the course of DKD. On the other hand, as expected and observed by other studies (21, 23), protein expression of fibrotic markers such as TGF-β1 and collagen IV was increased in both male and female BTBR ob/ob mice.

The importance of gender/sex to health and disease has gained more attention recently. Studies are emphasizing the complex crosstalk between sex hormones and the sex-dependent expression pattern of signals that can influence the course of renal injury development and severity (26, 27). Indeed, sexual dimorphism in kidney disease has been attributed to differences in sex steroid hormones. Most studies demonstrated a beneficial effect of estrogen and the deleterious effect of androgens (28, 29). Mechanistically, the sex disparities may be explained by differential regulation of inflammatory response, oxidative stress, among other effects (30).

Besides the hormonal effects, there is an increasing interest in the role that epigenetic modifications (such as DNA methylation) play in the phenotypic characteristics of diseases. It has been also constantly recognized that the development and progression of some diseases may be explained at the molecular level. Additionally, it has been shown that DNA methylation varies between males and females, which may underlie the sex biases observed in diseases (31). However, this field has much to be discovered and documented. Thus, considering that sex plays an important role in the epidemiology of diseases and that it has a strong influence on epigenetic modifications, studying the interplay between them is a necessary step. Considering this evidence, the focus of our study was to show an expression profile of key DNA (de)methylation enzymes under a diabetic kidney disease set and their differences between sexes in mice.

`Experimental models of diabetic kidney disease support the involvement of DNA methylation in renal cell functions and in the pathogenesis of DKD. However, data is still controversial and depends on the experimental model, the age of the animals, and the cell type studied. Zhang et al. (32), showed that 20 weeks male db/db mice have increased *Dnmt1* expression, without changes in *Dnmt3a* and *Dnmt3b*. The authors also demonstrated that albuminuria, glomerular hypertrophy, mesangial matrix expansion, and podocyte injury were attenuated after treatment with the DNA methylation inhibitor, 5-azacytidine. They proposed that inhibition of DNA methylation may be exploited as a new therapeutic target for protection against podocyte injury and thus treating diabetic nephropathy. On the other hand, as previously discussed, Oba et al. (24), showed that 15 weeks male db/db mice have decreased *Dnmt1* and *Dnmt3b* expression. Yang et al. (25) showed a time-dependent increase in DNMT3B and TET2 protein expression in male db/db mice.

By using another obese and type 2 diabetic model, the BTBR ob/ob mice, this study showed a decrease in *Dnmt1* gene expression due to the metabolic condition, both in males and females. On the other hand, *Dnmt3b* expression was independently modulated by sex. Whole kidney tissue gene expression of *Dnmt3a* was not changed, however glomerular protein expression of DNMT3A was modulated by sex, i.e., female WT mice showed increased basal expression compared to males. Additionally, DNMT3A was reduced in obese and diabetic females, showing that the metabolic condition also regulated its expression. Considering that DNMT3 activity is important for *de novo* DNA methylation, the modulation of its content may represent a relevant step for gene expression regulation in the development and progression of glomerular injury and DKD.

When studying global DNA methylation, it is important to explore not only DNMTs but also TETs, as the final status depends on the balance between the methylation and demethylation processes. The first studies correlating TET proteins expression with chronic kidney disease were performed by the group of Michael Zeisberg, in which they showed that TET3 was involved in the hypermethylation of *Rasal1* gene promoter, contributing to the activation of fibroblast and consequently to kidney fibrosis (33). Using the antifibrotic activity of bone morphogenic protein 7, low doses of hydralazine, and more recently, the CRISPR–Cas9 technology with designed constructs that directed TET3 to specific promoter sites, the researchers showed promising results of kidney fibrosis attenuation (34–36). As discussed earlier, Yang et al. (25) showed a time-dependent increase in TET2 expression in male db/db mice, without changes in subtypes 1 and 3 of TET. Interestingly, other studies in chronic kidney disease also observed increased TET2 expression (25, 37, 38).

The data presented here showed that metabolic condition reduced the transcript levels of all *Tet* genes, in male and female mice. Additionally, an independent effect of sex was observed for *Tet1* and *Tet3*. Despite the independent effects of metabolic condition and sex, when analyzing specifically the glomerular expression of these enzymes, it was noteworthy that TET1 and TET3 proteins were decreased only in BTBR ob/ob female mice, representing an interaction of these factors. The expression profile of these families of enzymes in the kidney tissue is an important first step to understanding the overall state of DNA methylation in DKD. However, as they are enzymes, further studies to deeply investigate their activities are necessary.

When studying epigenetics, many variables must be considered, including the species, the sex, the time of exposure to the insult, the tissue, and the cell populations. In fact, the differences between whole kidney tissue and glomerular content may partially be explained because changes in the DNA methylation status of the whole kidney are a summation of the changes in each individual cell population.

In conclusion, to our knowledge, this is the first study that shows a kidney profile of the expression of key (de)methylation enzymes, DNMTs and TETs, in the BTBR ob/ob experimental model and its association with sex. As epigenetic mechanisms have been increasingly recognized as key players in DKD, mostly mediating the expression of DKD-related genes and phenotypes, exploring differences between males and females is relevant. The detection of potential epigenetic factors during the course of DKD could be valuable for more precise treatments. Thus, the data presented here can enlighten the understanding of why males and females have different progression and severity of kidney disease. This knowledge opens new possibilities for using molecularly epigenetic therapy based on aberrant DNA methylation.

## Data availability statement

The raw data supporting the conclusions of this article will be made available by the authors, without undue reservation.

## Ethics statement

The animal study was reviewed and approved by Animal Use Ethics Committee of the Institute of Biomedical Sciences, University of São Paulo (numbers 5348280918 and 7358100320).

## Author contributions

BMVP and MCP collected samples, performed the studies, analyzedthe results, and wrote the manuscript; APML performed genotyping and RT-qPCR experiments; KT designed the study, provided funds and wrote the manuscript. All authors reviewed and approved the final version of the manuscript.

## References

[1] The Lancet. GBD 2017: A fragile world. Lancet (2018) 392(10159):1683. doi: 10.1016/S0140-6736(18)32858-7 30415747

[2] Foreman KJ Marquez N Dolgert A Fukutaki K Fullman N McGaughey M. Forecasting life expectancy, years of life lost, and all-cause and cause-specific mortality for 250 causes of death: Reference and alternative scenarios for 2016-40 for 195 countries and territories. Lancet (2018) 392(10159):2052–90. doi: 10.1016/S0140-6736(18)31694-5 PMC6227505 30340847

[3] Kalantar-Zadeh K Jafar TH Nitsch D Neuen BL Perkovic V. Chronic kidney disease. Lancet (2021) 398(10302):786–802. doi: 10.1016/S0140-6736(21)00519-5 34175022

[4] Kovesdy CP. Epidemiology of chronic kidney disease: an update 2022. Kidney Int Suppl (2011) (2022) 12(1):7–11. doi: 10.1016/j.kisu.2021.11.003 35529086 PMC9073222

[5] Sun H Saeedi P Karuranga S Pinkepank M Ogurtsova K Duncan BB. IDF diabetes atlas: Global, regional and country-level diabetes prevalence estimates for 2021 and projections for 2045. Diabetes Res Clin Pract (2022) 183:109119. doi: 10.1016/j.diabres.2021.109119 34879977 PMC11057359

[6] Badal SS Danesh FR. New insights into molecular mechanisms of diabetic kidney disease. Am J Kidney Dis (2014) 63(2 Suppl 2):S63–83. doi: 10.1053/j.ajkd.2013.10.047 PMC3932114 24461730

[7] Pereira BMV Katakia YT Majumder S Thieme K. Unraveling the epigenetic landscape of glomerular cells in kidney disease. J Mol Med (Berl) (2021) 99(6):785–803. doi: 10.1007/s00109-021-02066-2 33763722

[8] Wu T Ding L Andoh V Zhang J Chen L. The mechanism of hyperglycemia-induced renal cell injury in diabetic nephropathy disease: An update. Life (Basel) (2023) 13(2):539. doi: 10.3390/life13020539 36836895 PMC9967500

[9] Huang W Chen YY Li ZQ He FF Zhang C. Recent advances in the emerging therapeutic strategies for diabetic kidney diseases. Int J Mol Sci (2022) 23(18):10882. doi: 10.3390/ijms231810882 36142794 PMC9506036

[10] Del Castillo Falconi VM Torres-Arciga K Matus-Ortega G Díaz-Chávez J Herrera LA. DNA Methyltransferases: From evolution to clinical applications. Int J Mol Sci (2022) 23(16):8994. doi: 10.3390/ijms23168994 36012258 PMC9409253

[11] Tajima S Suetake I Takeshita K Nakagawa A Kimura H Song J. Domain structure of the Dnmt1, Dnmt3a, and Dnmt3b DNA methyltransferases. Adv Exp Med Biol (2022) 1389:45–68. doi: 10.1007/978-3-031-11454-0_3 36350506 PMC11025882

[12] Gerecke C Egea Rodrigues C Homann T Kleuser B. The role of ten-eleven translocation proteins in inflammation. Front Immunol (2022) 13:861351. doi: 10.3389/fimmu.2022.861351 35386689 PMC8977485

[13] Yin X Hu L Xu Y. Structure and function of TET enzymes. Adv Exp Med Biol (2022) 1389:239–67. doi: 10.1007/978-3-031-11454-0_10 36350513

[14] Thomas MC. Epigenetic mechanisms in diabetic kidney disease. Curr Diabetes Rep (2016) 16(3):31. doi: 10.1007/s11892-016-0723-9 26908156

[15] Smyth LJ Patterson CC Swan EJ Maxwell AP McKnight AJ. DNA Methylation associated with diabetic kidney disease in blood-derived DNA. Front Cell Dev Biol (2020) 8:561907. doi: 10.3389/fcell.2020.561907 33178681 PMC7593403

[16] Carper D Coué M Laurens C Langin D Moro C. Reappraisal of the optimal fasting time for insulin tolerance tests in mice. Mol Metab (2020) 42:101058. doi: 10.1016/j.molmet.2020.101058 32739449 PMC7471620

[17] Pereira BMV Thieme K de Araújo L Rodrigues AC. Lack of adiponectin in mice accelerates high-fat diet-induced progression of chronic kidney disease. Life Sci (2020) 257:118061. doi: 10.1016/j.lfs.2020.118061 32652137

[18] de Ponte MC Cardoso VG Gonçalves GL Costa-Pessoa JM Oliveira-Souza M. Early type 1 diabetes aggravates renal ischemia/reperfusion-induced acute kidney injury. Sci Rep (2021) 11(1):19028. doi: 10.1038/s41598-021-97839-7 34561469 PMC8463569

[19] Thieme K Pereira BMV da Silva KS Fabre NT Catanozi S Passarelli M. Chronic advanced-glycation end products treatment induces TXNIP expression and epigenetic changes in glomerular podocytes *in vivo* and *in vitro* . Life Sci (2021) 270:118997. doi: 10.1016/j.lfs.2020.118997 33453249

[20] Pandya Thakkar N Pereira BMV Katakia YT Ramakrishnan SK Thakar S Sakhuja A. Elevated H3K4me3 through MLL2-WDR82 upon hyperglycemia causes jagged ligand dependent notch activation to interplay with differentiation state of endothelial cells. Front Cell Dev Biol (2022) 10:839109. doi: 10.3389/fcell.2022.839109 35392173 PMC8982561

[21] Hudkins KL Pichaiwong W Wietecha T Kowalewska J Banas MC Spencer MW. BTBR Ob/Ob mutant mice model progressive diabetic nephropathy. J Am Soc Nephrol (2010) 21(9):1533–42. doi: 10.1681/asn.2009121290 PMC3013527 20634301

[22] Opazo-Ríos L Tejera-Muñoz A Soto Catalan M Marchant V Lavoz C Mas Fontao S. Kidney microRNA expression pattern in type 2 diabetic nephropathy in BTBR Ob/Ob mice. Front Pharmacol (2022) 13:778776. doi: 10.3389/fphar.2022.778776 35370692 PMC8966705

[23] Saad R Tadmor H Ertracht O Nakhoul N Nakhoul F Evgeny F. The molecular effects of SGLT2i empagliflozin on the autophagy pathway in diabetes mellitus type 2 and its complications. J Diabetes Res (2022) 2022:8337823. doi: 10.1155/2022/8337823 36313818 PMC9605841

[24] Oba S Ayuzawa N Nishimoto M Kawarazaki W Ueda K Hirohama D. Aberrant DNA methylation of Tgfb1 in diabetic kidney mesangial cells. Sci Rep (2018) 8(1):16338. doi: 10.1038/s41598-018-34612-3 30397232 PMC6218490

[25] Yang L Zhang Q Wu Q Wei Y Yu J Mu J. Effect of TET2 on the pathogenesis of diabetic nephropathy through activation of transforming growth factor β1 expression *via* DNA demethylation. Life Sci (2018) 207:127–37. doi: 10.1016/j.lfs.2018.04.044 29705354

[26] Ricardo AC Yang W Sha D Appel LJ Chen J Krousel-Wood M. Sex-related disparities in CKD progression. J Am Soc Nephrol (2019) 30(1):137–46. doi: 10.1681/asn.2018030296 PMC6317604 30510134

[27] Ziller N Kotolloshi R Esmaeili M Liebisch M Mrowka R Baniahmad A. Sex differences in diabetes- and TGF-β1-Induced renal damage. Cells (2020) 9(10):2236. doi: 10.3390/cells9102236 33023010 PMC7600610

[28] Seppi T Prajczer S Dörler MM Eiter O Hekl D Nevinny-Stickel M. Sex differences in renal proximal tubular cell homeostasis. J Am Soc Nephrol (2016) 27(10):3051–62. doi: 10.1681/ASN.2015080886 PMC5042665 27127188

[29] Darvishzadeh Mahani F Khaksari M Raji-Amirhasani A. Renoprotective effects of estrogen on acute kidney injury: The role of SIRT1. Int Urol Nephrol (2021) 53(11):2299–310. doi: 10.1007/s11255-020-02761-y 33458788

[30] Shen H Holliday M Sheikh-Hamad D Li Q Tong Q Hamad CD. Sirtuin-3 mediates sex differences in kidney ischemia-reperfusion injury. Transl Res (2021) 235:15–31. doi: 10.1016/j.trsl.2021.03.015 33789208

[31] Govender P Ghai M Okpeku M. Sex-specific DNA methylation: Impact on human health and development. Mol Genet Genomics (2022) 297(6):1451–66. doi: 10.1007/s00438-022-01935-w 35969270

[32] Zhang L Zhang Q Liu S Chen Y Li R Lin T. DNA Methyltransferase 1 may be a therapy target for attenuating diabetic nephropathy and podocyte injury. Kidney Int (2017) 92(1):140–153. doi: 10.1016/j.kint.2017.01.010 28318634

[33] Tampe B Tampe D Muller CA Sugimoto H LeBleu V Xu X. Tet3-mediated hydroxymethylation of epigenetically silenced genes contributes to bone morphogenic protein 7-induced reversal of kidney fibrosis. J Am Soc Nephrol (2014) 25(5):905–12. doi: 10.1681/asn.2013070723 PMC4005308 24480825

[34] Tampe B Tampe D Zeisberg EM Muller GA Bechtel-Walz W Koziolek M. Induction of Tet3-dependent epigenetic remodeling by low-dose hydralazine attenuates progression of chronic kidney disease. EBioMedicine (2015) 2(1):19–36. doi: 10.1016/j.ebiom.2014.11.005 25717475 PMC4337426

[35] Tampe B Steinle U Tampe D Carstens JL Korsten P Zeisberg EM. Low-dose hydralazine prevents fibrosis in a murine model of acute kidney injury-to-chronic kidney disease progression. Kidney Int (2017) 91(1):157–76. doi: 10.1016/j.kint.2016.07.042 27692563

[36] Xu X Tan X Tampe B Wilhelmi T Hulshoff MS Saito S. High-fidelity CRISPR/Cas9- based gene-specific hydroxymethylation rescues gene expression and attenuates renal fibrosis. Nat Commun (2018) 9(1):3509. doi: 10.1038/s41467-018-05766-5 30158531 PMC6115451

[37] Bao Y Bai M Zhu H Yuan Y Wang Y Zhang Y. DNA Demethylase Tet2 suppresses cisplatin-induced acute kidney injury. Cell Death Discovery (2021) 7(1):167. doi: 10.1038/s41420-021-00528-7 34226503 PMC8257623

[38] Wan F Tang YW Tang XL Li YY Yang RC. TET2 mediated demethylation is involved in the protective effect of triptolide on podocytes. Am J Transl Res (2021) 13(3):1233–44. PMC8014380 33841652

